# Pre-operative Ultrasound Localization for Removal of a Penetrating Foreign Body of Forearm: Technical Note

**DOI:** 10.7759/cureus.26940

**Published:** 2022-07-17

**Authors:** David Kirby, Daniel Seigerman

**Affiliations:** 1 Orthopedics, New York University (NYU) Langone Health, New York, USA; 2 Orthopedic Surgery, Rothman Orthopaedic Institute, New York, USA

**Keywords:** glass, penetrating wound, hand injury, surgical removal, ultrasound, foreign body

## Abstract

Retained or missed foreign bodies are a common complication associated with penetrating wounds. Ideal management includes immediate removal of the foreign material without any further damage to local tissues. However, removal is often difficult if the foreign body is small or has penetrated deeply. In this setting, the emergency room is a suboptimal environment for removal. Ultrasound utilized at the time of removal improved our ability to localize the foreign body, however this requires surgeon familiarity with the equipment. We describe the use of preoperative ultrasound guided foreign body localization with careful topographic skin marking to guide intraoperative foreign body removal with increased yield and obviating the need for ultrasound at the time of removal.

## Introduction

There are an estimated 11 million wounds treated annually in the US, with foreign bodies associated with 10% of these cases [1]. Of these, glass is one of the most common foreign bodies. The surgical exploration required can often lead to significant iatrogenic injury. This is especially true in the hand and forearm where complex anatomy and an abundance of vital structures can be challenging to those not well versed in the anatomy. Intra-operative ultrasound has been described as an effective method for guiding foreign body removal and limiting surgical dissection. However, this requires a surgical assistant familiar with the ultrasound and access to a sterile ultrasound in the operative field [2]. We have developed an alternative method for foreign body removal that allows for the benefits of ultrasound guidance without the need for ultrasound at the time of removal.

## Technical report

Radiographic evaluation

**Figure 1:**
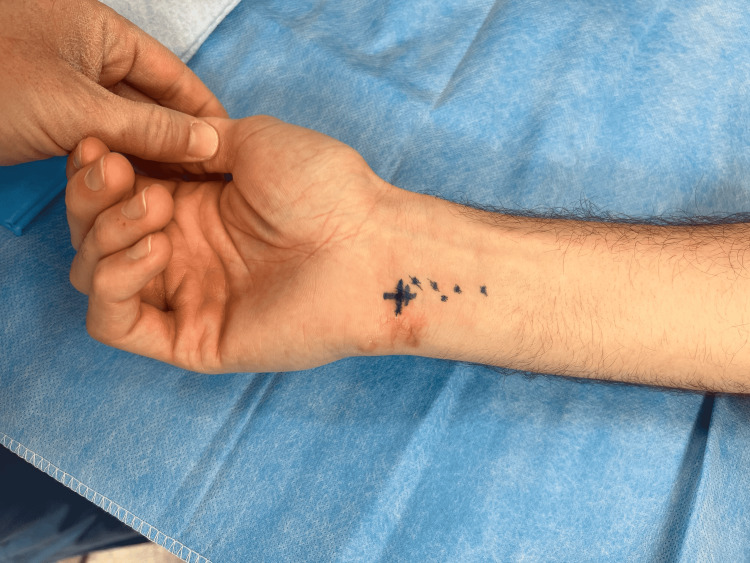
Ultrasound guided skin marking Blue marking over the wrist demonstrates the location of the foreign body. The ‘X’ is centered over the foreign body. This mark was initially made by the radiologist at the time of imaging and a photograph was sent to the surgeon. The mark was then recreated in preoperative area by the surgeon.

Patients should first be referred to a radiologist skilled in ultrasound techniques. This radiologist will draw an ‘X’ centered over the foreign body. It is essential that the ultrasound probe is centered directly over the foreign body with the probe perpendicular to the skin at the point which represents the shortest point of the foreign body to the surface of the skin. Notes should be taken on the depth, orientation, and number of fragments. A photo is then taken of the hand with the ‘X’ marking (Figure 1). If surgery is not planned to follow the ultrasound on the same day then a photo can be taken by the ultrasound technician which allows the surgeon to reapproximate the foreign body location on the day of surgery. The surgery should be planned to follow the ultrasound as soon as possible given that fragments can migrate within the forearm.

Indications/contraindications

This procedure is indicated in any patients undergoing surgical exploration for foreign body removal. In general, foreign body removal is indicated in patients with persistent pain due to foreign body, with concern of nerve, vessel or tendon injury, for those with cosmetic deformity related to the foreign body, and for those with an infection about the foreign body. Chronic foreign bodies can develop dense adhesions and thick fibrous tissue which may require an extensile approach obviating the need for ultrasound localization. This procedure is relatively contraindicated in patients with an asymptomatic foreign body.

Surgical technique

Using this skin marking and the known depth of the fragment, most smaller fragments can be quickly localized with a 1 cm incision. If the skin mark has faded, the photograph taken by the radiologist or radiology technician at the time of evaluation can be used to recreate the skin marking. If the initial puncture wound is in reasonable proximity, this should be incorporated into the incision. A single longitudinal incision is typically sufficient for foreign bodies within 0.5 cm of the skin surface however deeper foreign bodies may be missed if the incision parallels the fragment. In these cases with foreign bodies >0.5 cm deep, consideration should be made for a slight elliptical incision which creates a wider cone of dissection. The plane of dissection is extremely important as all ultrasound images and measurements were made with the probe perpendicular to the skin surface. Therefore dissection should be carried out center exactly over the X marking and perfectly perpendicular to the skin surface. Dissecting scissors or a hemostat are then used to carefully dissect the soft tissue, which additionally allows for the tactile metal-on-metal or metal-on-glass feel to help localize the foreign body. A hemostat or forceps can then be used to grasp and remove the foreign body. This technique allows for the localization and removal of exceedingly small fragments with excellent precision without the use of intraoperative ultrasound guidance. Foreign body removal is followed by copious irrigation and standard skin closure.

Expected outcomes

This technique limits the need for extensive surgical exploration and expedites surgery which also limits tourniquet time. This decreases the likelihood of iatrogenic injury to local tissues beyond what occurred during the initial trauma. Five to 10 minutes of tourniquet time is required for this procedure.

Complications

The complications of retained foreign bodies include infection, delayed healing, persistent pain, and late injury because of migration. Any surgical dissection incurs further risk of iatrogenic injury to local structures, including the nerves, vessels and tendons. Preoperative localization of the foreign body as well as meticulous dissection decrease the likelihood of these complications. Postoperatively, the wounds should be monitored for expected healing, without the need for antibiotic prophylaxis unless infection is suspected.

Limitations

This technique is highly dependent on a skilled ultrasound technician who is familiar with the technique.

## Discussion

Foreign body removal remains a precarious procedure given the complex anatomy of the hand. In a study of 65 patients with foreign bodies in the hand, only 30% of these patients were able to safely undergo foreign body removal at the time of presentation [3]. The study demonstrated that those who could not have the foreign body removed had significantly smaller foreign bodies, indicating that ability to locate the object likely played a role. Importantly, for those patients in whom the foreign body remained, only 4% developed persistent symptoms related to the foreign body [3]. Therefore, in the acute setting it is reasonable to remove the foreign body if easily accessible with a low threshold to leave the foreign body and address at a later time if the patient develops persistent symptoms.

Unfortunately, retained foreign bodies have become a target for malpractice claims, specifically in cases of untreated or undiagnosed foreign bodies [4]. Efforts should be made to diagnose foreign bodies in the acute setting. Radiographs have been shown to be particularly effective in the diagnosis of foreign bodies due to glass. One study in chicken legs demonstrated that radiographs have detection rates of 83% with 1 mm fragments and 99% with 2 mm fragments [4]. If radiographs are negative but concern for foreign body persists, ultrasound is a viable next step and has been shown to increase the sensitivity for diagnosing radiopaque objects and smaller glass fragments. A study of patients with concern for retained foreign body in the setting of negative radiographs found that ultrasound was able to identify the foreign body in 19 of 21 patients [5]. An additional benefit of ultrasound is that it provides a depth to guide in foreign body localization.

Ultrasound has been utilized both in the ED and in the operating room for identification and immediate removal of the foreign body [6,7]. An early case series described the use of ultrasound at the time of removal reporting successful removal of all foreign bodies which included glass, metal and wood [8]. In a series of 7390 patients, 99.5% of foreign bodies were able to be removed with an average operative time of 5 minutes using ultrasound [9]. Another study of 12 patients using ultrasound at the time of removal found success in 11/12 cases with an average operative time of 20 minutes. The one failed case was attributed to the depth of foreign body, which was 23 mm below the skin surface and in the thenar musculature [10]. No serious complications were reported in either of these studies. These studies demonstrate ultrasound as an effective tool at the time of removal, however the surgeon performing the foreign body removal must be familiar with sonography and comfortable with the equipment in the surgical field. Here we described a method that utilizes preoperative ultrasound with skin markings to guide surgical exploration without the need for ultrasound in the operative field. Safe and rapid removal of foreign bodies as small as 2 mm is possible.

## Conclusions

Foreign bodies in the hand can be difficult to remove in the acute setting due to the complexity of local anatomy and size of fragments. Ultrasound has improved our ability to localize foreign bodies, but use of the ultrasound by the surgeon in the operating room relays its own set of difficulties. Although dependent on an experienced ultrasound technician, by combining a preoperative ultrasound and topographic skin markings the surgeon can accurately do surgical exploration of the foreign body, greatly improving exploratory yield and limiting tissue dissection.
